# Roles for jasmonate- and ethylene-induced transcription factors in the ability of Arabidopsis to respond differentially to damage caused by two insect herbivores

**DOI:** 10.3389/fpls.2014.00407

**Published:** 2014-08-19

**Authors:** Erin M. Rehrig, Heidi M. Appel, A. Daniel Jones, Jack C. Schultz

**Affiliations:** ^1^Department of Biology and Chemistry, Fitchburg State UniversityFitchburg, MA, USA; ^2^Plant Sciences, Bond Life Sciences Center, The University of MissouriColumbia, MO, USA; ^3^Department of Biochemistry and Molecular Biology, Department of Chemistry, Michigan State UniversityEast Lansing, MI, USA

**Keywords:** Arabidopsis, herbivory, transcription factors, ERFs, ethylene, jasmonate, wounding

## Abstract

Plant responses to insects and wounding involve substantial transcriptional reprogramming that integrates hormonal, metabolic, and physiological events. The ability to respond differentially to various stresses, including wounding, generally involves hormone signaling and trans-acting regulatory factors. Evidence of the importance of transcription factors (TFs) in responses to insects is also accumulating. However, the relationships among hormone signaling, TF activity, and ability to respond specifically to different insects are uncertain. We examined transcriptional and hormonal changes in *Arabidopsis thaliana* after herbivory by larvae of two lepidopteran species, *Spodoptera exigua* (Hübner) and *Pieris rapae* L. over a 24-h time course. Transcriptional responses to the two insects differed and were frequently weaker or absent in response to the specialist *P. rapae*. Using microarray analysis and qRT-PCR, we found 141 TFs, including many AP2/ERFs (Ethylene Response Factors) and selected defense-related genes, to be differentially regulated in response to the two insect species or wounding. Jasmonic Acid (JA), JA-isoleucine (JA-IL), and ethylene production by *Arabidopsis* plants increased after attack by both insect species. However, the amounts and timing of ethylene production differed between the two herbivory treatments. Our results support the hypothesis that the different responses to these two insects involve modifications of JA-signaling events and activation of different subsets of ERF TFs, resulting in different degrees of divergence from responses to wounding alone.

## Introduction

Plant responses to insects and wounding are complex, involving differential perception, multiple signaling pathways, and extensive transcriptional reprogramming (Delessert et al., 2004; DeVos et al., 2005; Rehrig et al., 2011). Perception of insect attack by plants is thought to occur at the site of herbivory via damage- or herbivore-associated molecular patterns (DAMPS or HAMPs, respectively). DAMPs are plant-derived signals produced after wounding and include ATP, Volatile Organic Compounds or elicitors in cell wall fragments (reviewed by Kaku et al., 2006; Alborn et al., 2007; Heil, 2009; Pieterse et al., 2012). Plants also respond to HAMPs found in insect oral secretions (OS) including fatty-acid/amino acid conjugates and B-glucosidase (Mattiacci et al., 1995; Pare and Tumlinson, 1999; Schmelz et al., 2006; Wu and Baldwin, 2009). Insect feeding patterns may further influence this response (Wittstock et al., 2004; McCartney, 2007). Although the mechanisms are uncertain, it is suggested that plants can identify their attacker and activate species-specific defenses (DeVos et al., 2005; Mewis et al., 2005; Vogel et al., 2007; Galis et al., 2009; Stork et al., 2009). Yet, how hormone concentrations and other important components of plant signaling pathways are interpreted and integrated by the plant to activate appropriate responses after wounding or herbivory by different insects remains unclear.

The plant hormones jasmonic acid (JA), salicylic acid (SA), abscisic acid (ABA), and ethylene (ET) are among the critical players in the events following abiotic and biotic stress, including insect attack. Hormones also appear to modulate the fine-tuning of defenses in response to different insects (Reviewed by Zhu-Salzman et al., 2004; DeVos et al., 2005; Mewis et al., 2005; Thompson and Goggin, 2006; Wasternack and Hause, 2013). The mechanisms of pathway control and integration in plant immunity also involve the hormones ABA (Abe et al., 2003; Bodenhausen and Reymond, 2007), auxin (Grunewald et al., 2009), gibberellic acid (GA) (Hou et al., 2010), and cytokinins (Choi et al., 2011) which interact with stress hormones in a complex network to maximize survivorship of the plants (reviewed by Pieterse et al., 2012). Some of the first signaling events following perception of insect attack are the rapid accumulation and transport of JA and a quick “burst” of ET production (reviewed by Reymond and Farmer, 1998; Winz and Baldwin, 2001; Thaler et al., 2002; Babst et al., 2005; Wu and Baldwin, 2009). The release of ethylene after wounding and herbivory depends on plant species being attacked and the herbivore species involved but is generally greater in response to herbivory than to wounding (Von Dahl and Baldwin, 2007). Von Dahl et al. (2007) hypothesized that ethylene may attenuate or fine-tune responses based on its interaction or cross talk with other phytohormones. Larvae of *Pieris rapae* grew poorly on mutant plants with compromised ethylene signaling, which was associated with an increase in JA-inducible indolyl glucosinolates (Mewis et al., 2006). Studies conducted by Bodenhausen and Reymond (2007) and Stotz et al. (2000) showed that *Arabidopsis* plants defective in ethylene signaling had increased resistance to *Spodoptera littoralis*. On the other hand, DeVos et al. (2006) found that ethylene production after *P. rapae* feeding primed plants for increased resistance to future viral infection.

It is well-established that JA and its amino acid conjugate, JA-isoleucine (JA-IL) are critical players in plant responses to insects as well as wounding (Chini et al., 2007; Thines et al., 2007). Plants with mutations in the JA signaling pathway such as *coi1* and *jar1* have increased susceptibility to insect attack (Thaler et al., 2002; Mewis et al., 2005; Bodenhausen and Reymond, 2007; Verhage et al., 2011). ET modulates JA-signaled defenses (Lorenzo et al., 2004) and SA often inhibits them (Beckers and Spoel, 2006). One goal of this study was to elucidate how the interactions among JA, SA, and ET production may influence transcriptional responses to attack by different insects.

Transcription factors (TFs) provide a likely mechanism for translating hormone signaling into the subsequent activation of defense genes differentially expressed in response to attack by different insect species. For example, the activation of TFs in the WRKY and APETALA2/ETHYLENE RESPONSE FACTOR (AP2/ERF) families has been shown to be required for differential responses *in planta* to JA treatment, pathogenesis, herbivory, and wounding (Delessert et al., 2004; Reymond et al., 2004; Lu et al., 2011). Additional transcriptional responses to wounding, JA treatment, or JA and ET often include the up-regulation of genes involved in plant defense such as *PDF1.2, VSP2, LOX2*, and chitinases (reviewed by Boter et al., 2004; Bodenhausen and Reymond, 2007; Pieterse et al., 2012; Wasternack and Hause, 2013), which are largely under transcriptional control of ERFs and the ABA/JA responsive-TF, MYC2 (reviewed by Brown et al., 2003; Lorenzo and Solano, 2005; Dombrecht et al., 2007; reviewed by Wasternack and Hause, 2013). Because ERFs, members of the MYC family and other TFs serve as points of cross talk between the JA, ET, and SA hormone pathways (Li et al., 1999; Abe et al., 2003; Lorenzo et al., 2003; Yadav et al., 2005; Robert-Seilaniantz et al., 2011; Verhage et al., 2011; Schweizer et al., 2013), they are likely to be critical players in responses to different biotic stresses, including insect herbivory.

Using microarray analysis and qRT-PCR, we identified and quantified transcripts whose expression in *Arabidopsis thaliana* (Columbia) was altered in attacked (local) and unattacked (systemic) tissues after feeding by *S. exigua* and *P. rapae*. We hypothesized that the two insects elicit different patterns of hormone production, differential expression of TF genes, and differential expression of selected defense-related genes. We identified several unique transcriptional patterns within the AP2-ERF gene family and associated this with both increased JA and ET elicitation and differential regulation of defense-related gene expression at different times after feeding by the 2 different insects.

## Materials and methods

### Plants and insect care

Eggs of the caterpillar *Spodoptera exigua* Hübner (Noctuidae) were obtained from Benzon Research (Carlisle, PA, USA) and larvae were reared on artificial diet (Bioserv, Frenchtown, NJ, USA). The caterpillar *Pieris rapae* L. (Pieridae) was maintained as a culture on pak-choi and originated from the Carolina Biological Supply Company (North Carolina). Both caterpillar species were transferred to *Arabidopsis thaliana* (L.) ecotype Columbia (Col-0) plants 1 day before the experiments to acclimate to the new host. Col-0 seeds were vernalized in 2% agar and sown into 6 × 5 cm pots containing sterile Metromix 200 soil (Sun Gro Horticulture) and Osmocote. Plants were chamber grown at 22 ± 1°C, 65 ± 5% relative humidity, and 200 μmol m^−2^s^−1^ light intensity on a short-day (8:16 (L:D)) photoperiod to delay flowering and keep plants in the rosette stage. Plants were watered as needed.

### Insect and wounding treatments

Insect treatments for the microarray analysis were conducted as described by Appel et al. (2014), (this edition). Control plants were grown in the same conditions as insect-treated plants except cages without insects were placed on them. Experiments were designed to minimize circadian influences and capture gene expression in full-rosette leaves. Two to three second- and third-instar *S. exigua* and *P. rapae* caterpillars were allowed to feed on 5–6 week old plants until 20–30% of the leaf area of 4 leaves was removed. Caterpillars were kept in custom-made soft cages, which were checked periodically for plant damage. To maximize damage in minimal time (<30–45 min), caterpillars that were not eating were replaced with ones that were more cooperative. Furthermore, caterpillars were withheld food for 24 h to encourage eating. This technique was used so that we could observe early responses to caterpillars, which would be missed if an aggressive time line were not imposed. For example, plants with insects imposing 20–30% damage quickest were used for the 15-min and subsequent samples, respectively. Control and treatment plants were kept in the same experimental area. Experiments with each insect were conducted on separate days and were repeated 3 times. Pictures of damaged and control plants can be found in the Supplementary Material. Once sufficient damage was achieved, caterpillars were removed and the plants were returned to the growth chamber until processed for RNA or hormone analysis. For wounding treatments, insect damage was simulated using a sterile damage wheel across both sides of the mid-rib on 6 leaves. Leaves were harvested for gene expression or hormone analysis beginning at 15 min up until 48 h after cage removal or wounding. Four plants were used for each bioreplicate and four bioreplicates were collected per treatment. We harvested the four treated leaves on each plant for different assays: two for RNA tissue, one for JA/SA measurement, and one for an initial ethylene analysis. RNA and JA/SA sample leaves were weighed, flash frozen in grinding tubes immersed in liquid nitrogen, and then stored at −80°C. Ethylene samples were processed immediately.

### Microarray analysis and transcription factor identification

Plants were harvested and RNA was isolated for microarray analysis as described by Appel et al. (2014). Briefly, RNA was isolated using the TRIZOL method (Invitrogen, Carlsbad, CA, USA) and its quality was determined by an Agilent 2100 Bioanalyzer. RNA was reverse transcribed into labeled cDNA using a T17 primer, dNTPs and Cyanidin-3 and 5 dUTPs. cDNA was purified and hybridized to an Operon v1 microarray chip with 26,090 *Arabidopsis* gene specific 70-mer oligonucleotides. Four replicate chips were used for each treatment. Analysis of the data, including statistics and identification of false positives was done using the methods described by Ehlting et al. (2005, 2008), Storey and Tibshirani (2003), and Pylatuik and Fobert (2005). To analyze the microarray data for putative TFs, we conducted a literature search and used the online databases, GenBank, Gene Annotation tool (GO) from the TAIR website (www.Arabidopsis.org) and DATF (Database of *Arabidopsis* TFs; Guo et al., 2005). A complete list of all Transcription Factor gene names and abbreviations can be found in Table S3.

### Gene expression via real time qRT-PCR

The expression of ERFs, *MYC2 (JIN1*), Housekeeping genes, and 6 defense-related marker genes was measured by semi-quantitative Real-Time PCR. A list of genes and primers can be found in Table S1. Total RNA from insect-attacked and control tissue samples was extracted using Sigma Total Plant RNA kits (STRN50, St. Louis, MO, USA) or the TRIZOL method (microarray experiment only). The same RNA used for the microarray was used for qPCR for the initial ERF analysis to confirm the array results. Otherwise, RNA was isolated from insect-treated plant material from subsequent experiments. RNA quality was confirmed using a Bio-Rad Experion automated electrophoresis system (Hercules, CA, USA) and a Bio-Rad RNA standard sensitivity kit which adequately detects and quantifies nanogram levels of RNA.

Primers were designed and tested using methods described in Rehrig et al., 2011. We used Primer 3 Software (Rozen and Skaletsky, 2000) and Invitrogen's Vector NTI Software (Carlsbad, CA, USA) as well as IDT's on-line tool, OligoAnalyzer for further prediction of primer dimers. All primers were BLASTed in NCBI to ensure specificity of amplification. We performed gel electrophoresis of PCR products and detected single bands of expected size. Additionally, melting curve analysis of all PCR products was done *via* real-time PCR. All PCR products were sequenced to ensure that only gene products of interest were being amplified.

We treated samples with Turbo DNAse (Ambion, Austin, TX, USA) according to the manufacturer's specifications. RNA quantity after DNAse treatment was measured using a NanoDrop (ThermoScientific, Wilmington, DE, USA) in triplicate for each sample immediately before the reverse transcription reaction. We followed the protocol for Invitrogen's Superscript III 2-step qRT-PCR kit with Platinum SYBR Green qPCR Super-Mix UDG (Carlsbad, CA, USA) with minor modifications. To acquire sufficient amounts of cDNA for all of the subsequent real time PCR reactions, 4 reverse transcription reactions were performed for each RNA sample. These were done in 96-well plates and the volumes of 4 technical replicates for each sample were pooled, sub-sampled for a standard curve mix, and diluted 5×.

All PCR reactions were run in 96-well plates. Each bioreplicate was run in triplicate. Five mL of cDNA template, 5 mM primer pair mixes, molecular-grade water, and Platinum SYBR Green for a total of 20 mL was used for PCR. Amplification was then conducted under the following conditions on a MJ Research Opticon 2 DNA Engine: 50°C UDG treatment for 2 min, 95°C denaturation for 2 min, followed by 40 cycles of 95°C denaturation for 15 s, 56°C annealing for 30 s and 72°C extension for 30 s. After extension, but prior to fluorescence measurement reads, the temperature was ramped to approximately 1.5–2.0°C below the gene product melting curve start (Tm, –dl/dT min). A final 5 min extension at 72°C followed by a complete melting curve analysis from 72 to 95°C were then conducted.

### Data analysis of qRT-PCR data

qRT-PCR data were acquired using the standard curve method (Larionov et al., 2005). All data were initially analyzed using Opticon 3 Monitor Software. We used LinReg PCR (Ramakers et al., 2003) to identify a value for the threshold of fluorescence. We entered this value into the Opticon Software Program, which automatically calculated expression values from the Ct values based on the regression equation of the standard curve. Expression values for 24-h data from RNA from the microarray analysis were normalized against the geometric mean of 18S and G6PD5. Because a suitable housekeeping (HK) gene could not be found for the 6-h data, all 6-h expression levels were normalized to the total amount of cDNA in the PCR reaction using a correction factor. Because we had experimentally demonstrated that HK genes were inappropriate normalization factors for measuring gene expression after insect attack, later experiments with defense-related gene expression were normalized to the expression of an exogenous Luciferase RNA spike added prior to reverse transcription (Rehrig et al., 2011). Outliers for qRT-PCR and hormone measurements were identified using a one-pass Extreme Studentized Deviate (ESD) test (Pillai and Tienzo, 1959) and eliminated from the analyses. Statistically significant differences in final gene expression ratios between treatments and controls for both the *P. rapae* and the *S. exigua* experiment were identified using the PROC NPAR1WAY command in SAS and Kruskal-Wallis analyses (SAS Institute, Cary, NC, USA). Gene expression data displayed in **Figure 6** were transformed using the Log^2^ values of fold changes. A Hierarchal cluster analysis was done using the Spearman Rank Correlation feature in the software Cluster 3.0 (Eisen et al., 1998) and monitored with Java TreeView 1.1.3 (Saldanha, 2004).

### Ethylene measurements

For ethylene analysis, additional experiments were conducted using similar methods as described except the time course was extended to include the original time points (15 min, 30 min, 1 h, 2 h, 6 h, 24 h) as well as 12, 36, 48, and 72 h treatments. Four leaves from 1 plant (1 bioreplicate) from either insect-attacked or control plants were placed in sealed 10cc glass vials and allowed to incubate for 30–90 min. Four bioreplicates were taken for each treatment. Air was then drawn off using a 5cc syringe and manually injected into an HP Gas Chromatograph. ET levels were calculated using a regression equation of a standard curve and corrected for fresh weight and incubation time. To identify differences in ethylene, JA, JA-IL, and SA levels among treatments, we conducted an ANOVA in SAS (Cary, NC, USA). Statistically significant differences between treatments at a *p*-value of 0.05 or lower were determined using the PROC GLM command and *post-hoc* Tukey values.

### JA and JA conjugate measurements

JA and JA-IL levels were quantified using an ethyl acetate extraction method in conjunction with HPLC/MS similar to that described in Chung et al. (2008). Briefly, samples (approximately 150 mg tissue) were frozen in liquid nitrogen and hormones were extracted using 1 mL of extraction solvent (80:20 methanol:water + 0.1% formic acid) for 18 h at −20 C. Extracts were then centrifuged (10,000 × *g* for 10 min at 4°C) and the supernatant was transferred to autosampler vials. Five μL of each supernatant were injected into a Waters UPLC BEH C18 column (2.1 × 50 mm; 1.7 μm particles) held at 50°C on a Waters (Milford, MA, USA) Acquity ultraperformance liquid chromatography (UPLC) system that was coupled to a Waters Quattro Premier XE tandem quadrupole mass spectrometer. Separation was performed using a linear gradient based upon 0.15% aqueous formic acid (A) and methanol (B) over a 3-min program using a total flow rate of 0.4 mL/min. Quantification of JA and SA was performed using electrospray ionization in negative-ion mode using multiple reaction monitoring (MRM), using *m*/*z* 209 ≥ 59 for JA, *m*/*z* 322 ≥ 130 for JA-IL (Chung et al., 2008), and *m*/*z* 137 ≥ 93 for SA (Zeng et al., 2011). Peak areas were integrated, and calibration curves generated, using Waters QuanLynx software.

### Insect feeding and glucosinolate assays

Insect feeding bioassays with *P. rapae* and *S. exigua* were conducted separately. Insects were weighed before and after feeding. One insect was placed on one plant (*N* = 12−31) of either WT or *erf104, erf105, erf5, erf6* mutant plants (Shuqun Zhang, personal communication) and enclosed using customized plastic cages with mesh lids. Control plants from each genotype were also enclosed in cages, but received no insect treatments. Plants were placed under growing lights under 12 h days and insects were allowed to feed for 24–48 h, leaving some tissue for GS analysis. Growth rates were calculated according to actual time spent feeding on the plants. The performance of the insects was determined by a suite of nutritional indices that describe the consumption, growth, and efficiency with which food is converted to growth (Slansky and Scriber, 1985). Relative Growth Rates (RGR), Relative Consumption Rates (RCR), and Efficiency of Conversion Indices (ECI) were calculated (see Supplementary Material). Any insects that died, pupated or molted during the experiment were eliminated from the analysis. The amount of tissue eaten was determined using a digital phenotyping protocol described by Green et al. (2012). Total indolyl and aliphatic glucosinolate content in the remaining tissue was measured using a method described by Mewis et al. (2006). Insect-attacked plants were used to determine induced GS levels, while control plants served as baselines for constitutive levels.

## Results

### Overall patterns of transcription factor expression in response to insects or wounding

Of the approximately 1500 putative TFs in the *Arabidopsis* genome (Riechmann et al., 2000), we identified 141 genes encoding TFs whose expression was statistically significantly altered by wounding, *P. rapae* or *S. exigua* attack (Table S2). The TFs differentially expressed in response to insects or wounding represented 25 of the 50 families characterized in the AgrisTF Database (Davuluri et al., 2003). Families with the most members represented were the AP2-ERF/RAV (18), MYB (18) Homeobox (11), bHLH (10), and NAC (9) as well as ZIM-Related Proteins/JAZ (7). The two caterpillars and wounding elicited different expression patterns of TFs (Table S4). This is most prominently seen in the AP2/ERF Family where only *RAV2* was affected by both insects, and its expression was increased by *S. exigua* feeding and decreased by *P. rapae* feeding. Similarly, *ZAT10, ZAT5, ZAT12, WIP4*, and *AZF3*, members of the C2H2 transcription factor family, were up-regulated by *S. exigua* or wounding, but not by *P. rapae* in any tissue or treatment. Only wounding caused a change in the expression of *LOB* genes, which are involved in organ development (Husbands et al., 2007). Genes in JAZ family, which are well-documented to be JA-responsive (Chung et al., 2008) were widely elicited by both insects and wounding. Although JAZ proteins are not true TFs, they strongly interact with MYC2 and other important transcriptional regulators such as NINJA and TPL within the nucleus to control the expression of JA-induced genes (Pauwels et al., 2010). Therefore, they were included in our analysis.

We confirmed the contrasts for AP2/ERF TF genes with qRT-PCR on 17 of the 24 affected AP2/ERF TFs (including 2 RAV genes). We were able to statistically validate 20/26 of the microarray expression values for the ERFs; and, if we include all instances where qRT-PCR values were in the same direction as those of the microarray (i.e., similar to Northern blots), we achieved over 92% confirmation (Table 1). qRT-PCR data in conjunction with array data clearly show that *TINY2* is responsive to only *P. rapae*, whereas *SIMRAP2.4, ORA47, ERF11*, and *ERF104* were solely responsive to *S. exigua* feeding.

**Table 1:**
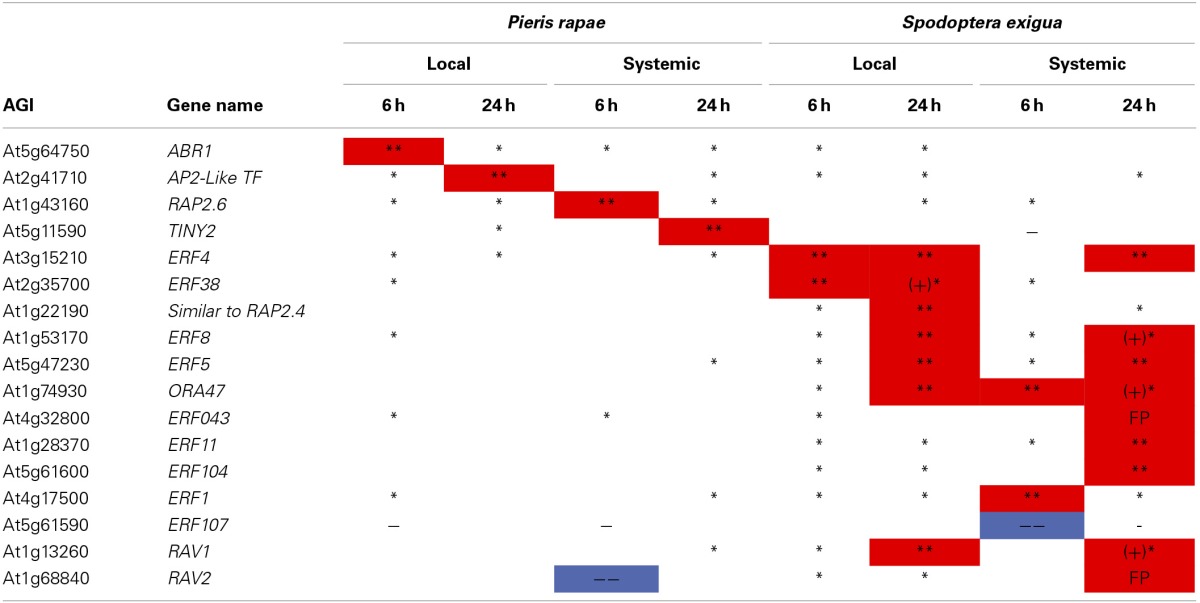
**qRT-PCR confirmation of AP2-ERF transcription factor genes identified by microarray analysis-ERF transcription factor genes affected by *P. rapae* and *S. exigua* in the array were amplified and quantified using qRT-PCR**.

There were 26 cases in which AP2-ERF genes were significantly up- or down-regulated in the microarray, noted by red (up) or blue (down) coloring. We confirmed 20 of these expression values using qRT-PCR, which are indicated by double asterisks (up) or double minus (down). Two false positives (FP) were detected. Single asterisks (up-regulation) or minus signs (down-regulation) represent expression level changes that were found to be significant (p < 0.05) through qRT-PCR but not by the array. The symbol “(+)*” indicates an unconfirmed positive where fold change measured by qRT-PCR was directionally similar to the array, but not significantly different from controls.

### Insect elicitation of ethylene release

**Figure 1:**
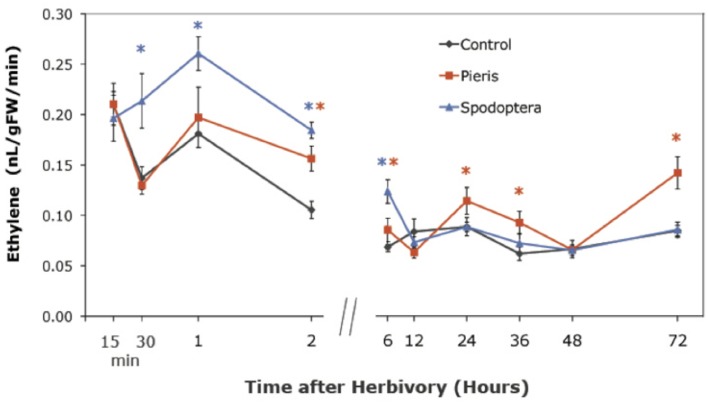
**Ethylene production in WT *Arabidopsis* plants after short-term *P. rapae* and *S. exigua* feeding over a 72-h time course-Ethylene was measured by Gas Chromatography as nanoliters/gram fresh weight/minutes of incubation time**. Asterisks represent data points that are significantly different than controls as determined by the GLM method in SAS (*p* < 0.05).

The TF expression results led us ask whether insects induce ethylene production as a potential signaling mechanism in *Arabidopsis*. We used gas chromatography (GC) to measure ethylene levels emitted by locally attacked tissue at selected time points. Both *P. rapae* and *S. exigua* feeding induced the production of ethylene, but the times when levels became significantly different from controls differed. *S. exigua* induced significantly higher levels of ethylene than controls in *Arabidopsis* tissue by 30 min, while ethylene production by *P. rapae* -attacked plants did not differ from controls until after 2 h (Figure 1). Moreover, *P. rapae*-induced ethylene production remained higher than controls at later time points. Our results indicate that ethylene production induced by *S. exigua* occurs as a rapid burst shortly after the insect feeds on the plant, while ethylene production after *P. rapae* feeding is delayed. Ethylene levels in our analysis are lower than what has been typically seen with necrotroph and pathogen infestation (Penninckx et al., 1998; DeVos, 2006; Mur et al., 2009) or compared with other herbivore studies (DeVos et al., 2005). This may be due to our sampling technique in which damaged leaves were kept on the plants until their harvest periods during the time course. Therefore, ET was not allowed to accumulate in the headspace of the collection vials during the recovery period after herbivore removal.

### Insect elicitation of jasmonic acid, jasmonic acid-isoleucine, and salicylic acid

**Figure 2:**
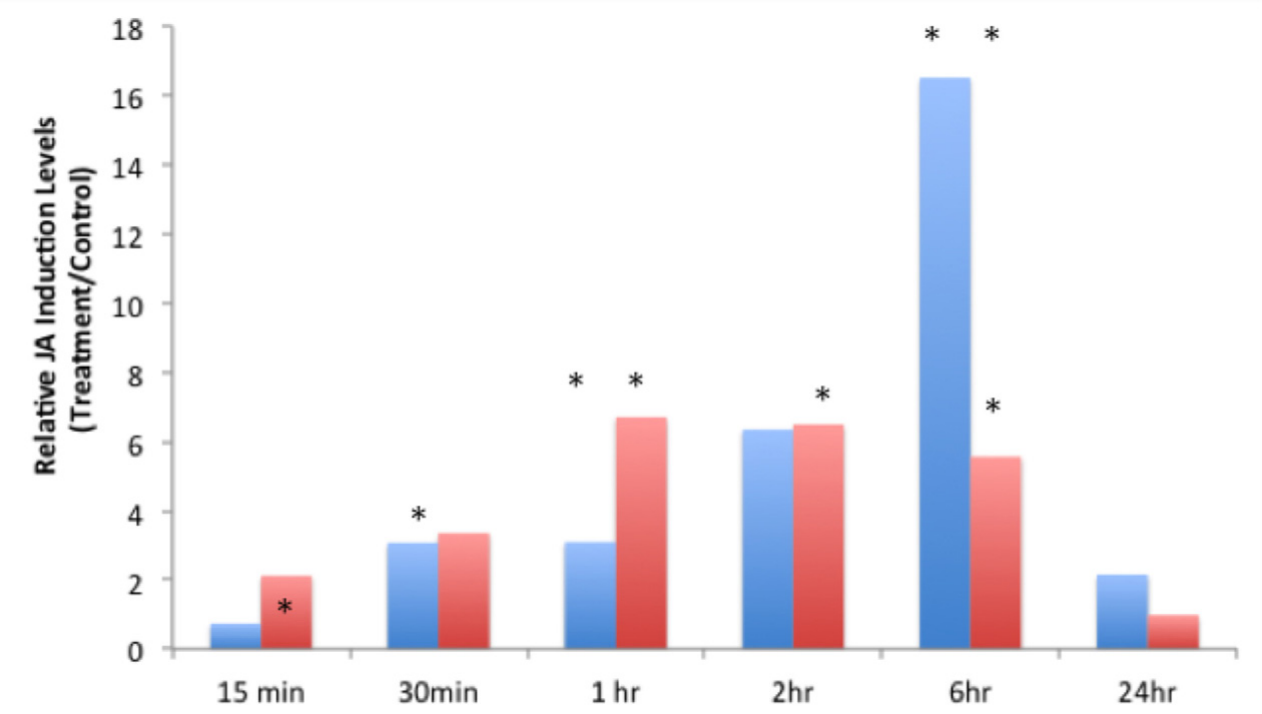
**Relative Jasmonic Acid levels in WT *Arabidopsis* plants after *S. exigua* and *P. rapae* feeding over a 24-h time course-JA was measured by UPLC-MS/MS as pmol/g fresh weight**. Blue bars represent JA levels in *S. exigua* treatments/controls. Red bars in represent JA levels in *P. rapae* treatments/controls. Asterisks represent time points where JA levels in the treatment plants were significantly greater than controls as determined by the GLM method in SAS (*p* < 0.05).

**Figure 3:**
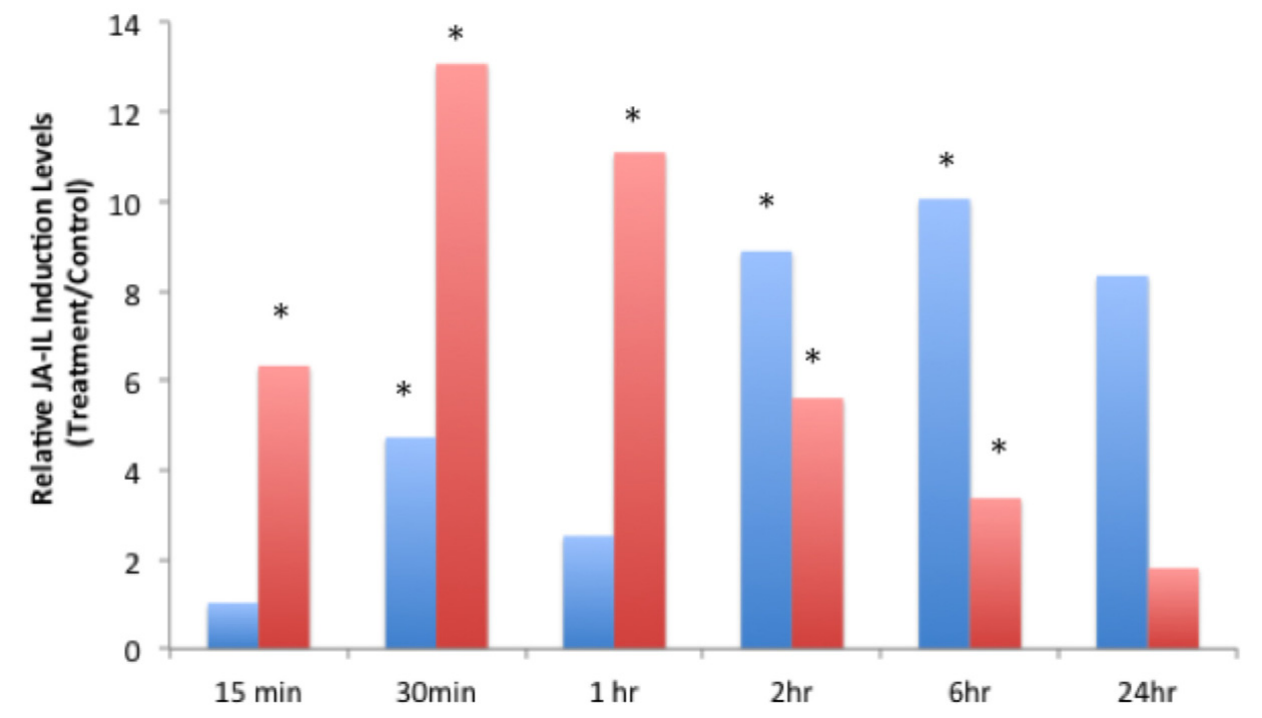
**Relative Jasmonic-isoleucine (JA-IL) Acid levels in WT *Arabidopsis* plants after *S. exigua* and *P. rapae* feeding over a 24-h time course-JA-IL was measured by UPLC-MS/MS as pmol/g fresh weight**. Blue bars represent JA-IL levels in *S. exigua* treatments/controls. Red bars in represent JA-IL levels in *P. rapae* treatments/controls. Asterisks represent time points where JA-IL levels in the treatment plants was significantly greater than controls as determined by the GLM method in SAS (*p* < 0.05).

JA-IL and SA levels were measured using UPLC-MS/MS. We found no significant increases in SA in response to insect treatments (Supplementary Material, Figure 1). However, relative levels of JA (Figure 2) and JA-IL (Figure 3) in insect-treated plants as compared to controls varied between time points. Absolute levels of JA and JA-IL induction can be seen in the Supplementary Material (Figures S2, S3). *S. exigua* elicited a statistically significant increase in JA above controls at 0.5, 2, and 6 h after feeding. The higher mean at 1 h was not statistically significant, probably due to the larger standard deviation at this time point (*p* = 0.1104). In response to *P. rapae*, JA levels increased significantly immediately (after 15 min) and remained above controls until 24 h after treatment. Patterns of JA-IL production after *S. exigua* and *P. rapae* feeding matched those of JA.

### ERF and defense gene expression

**Figure 4:**
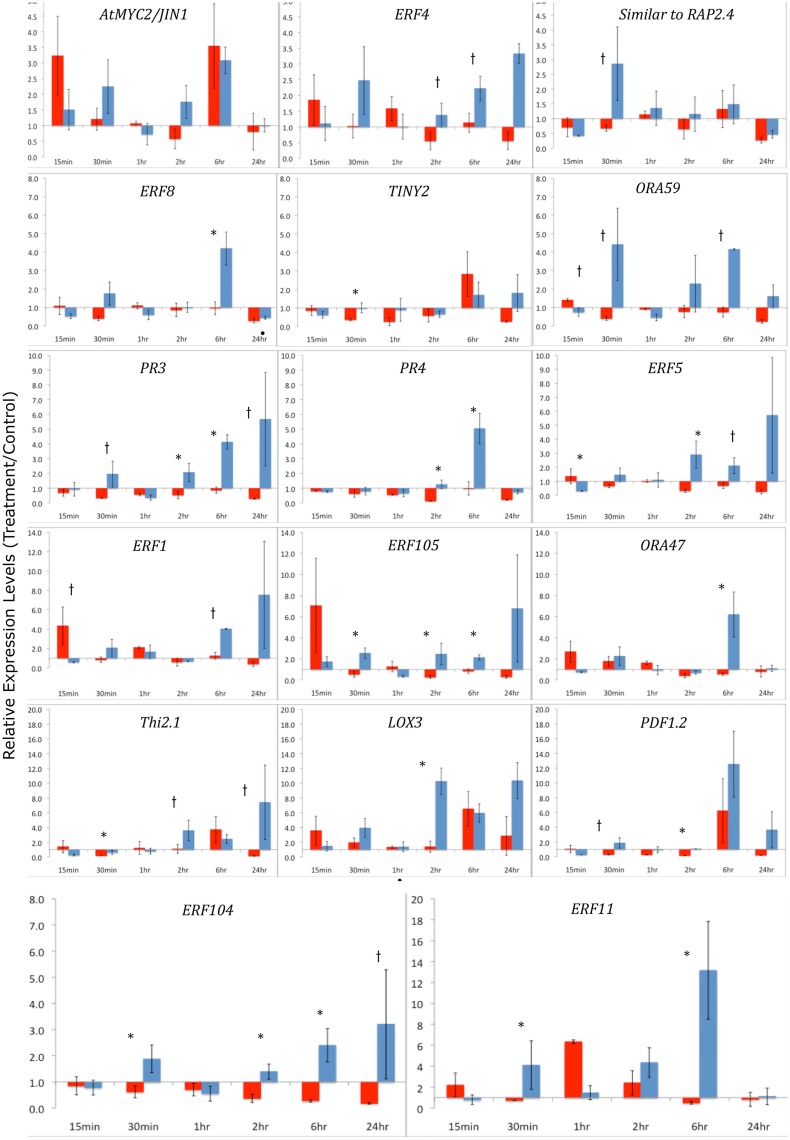
**qRT-PCR of ERF Transcription Factors and defense-related genes after Herbivory-Y-axes represent fold changes of treatment/controls**. Arabidopsis leaf tissue was collected 15 min, 30 min, 1 h, 2 h, 6 h, and 24 h after herbivory by *S. exigua* or *P. rapae* caterpillars. Red bars indicate *Pieris* treatments and blue bars represent *Spodoptera* treatments. qRT-PCR data were normalized to RNA quantity and the expression of an exogenous LUC spike. Fold changes differ in scale between each row. Control plants (cage only, no insects) were paired with treatment plants. Error bars represent the standard error of the means of the bioreplicates for each treatment and time point. Asterisks (*p* < 0.05) and lower case †'s (*p* < 0.08) indicate statistically significant differences between insect treatments as determined by Kruskal–Wallis ANOVA Analyses for ratios.

**Figure 5:**
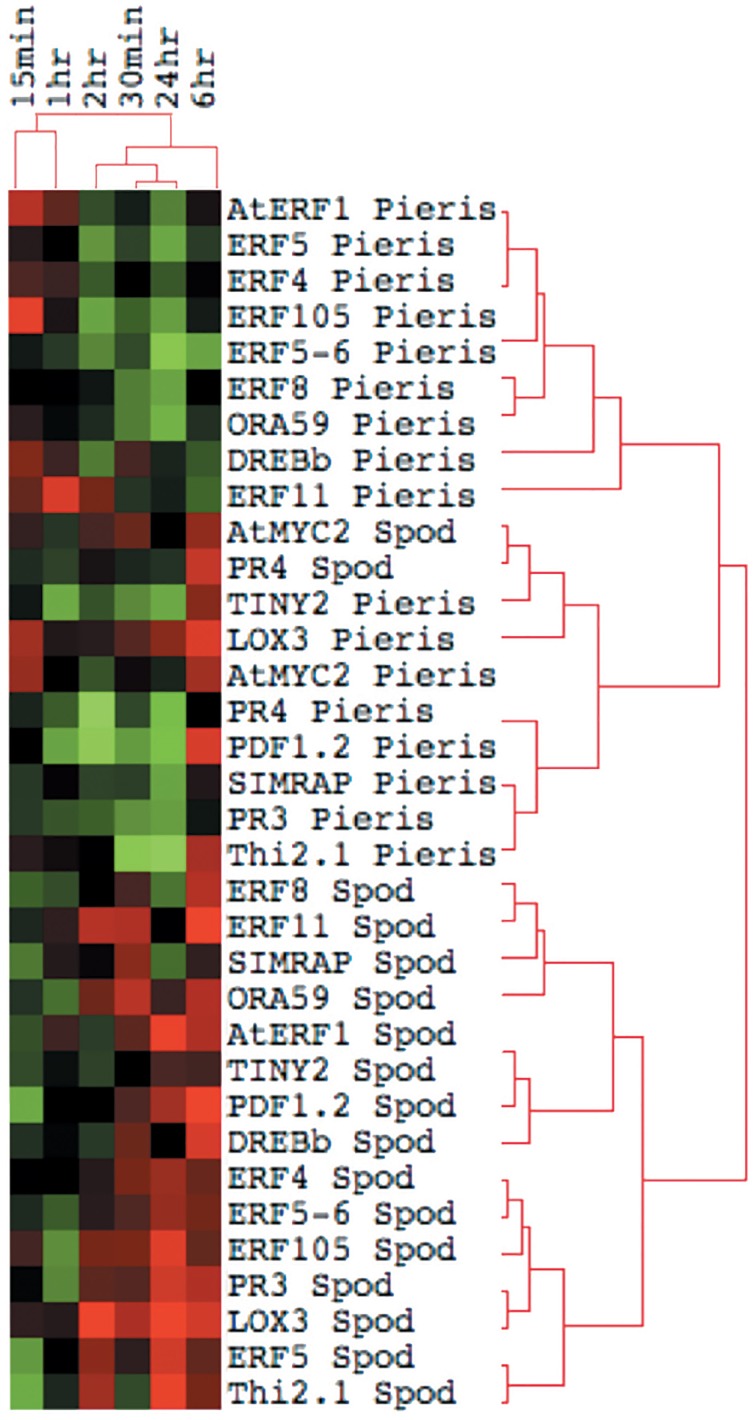
**Cluster Analysis and Heat Map of Defense-Related Gene Expression by *P. rapae* vs. *S. exigua***. Gene expression values in Arabidopsis tissue after insect treatments were and clustered by time after feeding using the method described by Eisen et al. (1998). Red color indicates the up-regulation of a gene, while green shows down-regulation. A black box represents no change in expression.

To further understand the role of ET and JA signaling in response to feeding by caterpillars of the same two lepidopteran species, we measured differences in the expression of genes encoding ERF TFs and defense-related genes. We monitored gene expression patterns of *ERF*s and down-stream defense genes through time (Figure 4). Plants exposed to *S. exigua* feeding showed dramatic transcriptional responses. In general, the expression of both *ERFs* and defense-related genes was greater after feeding by *S. exigua* than by *P. rapae*. Only three TFs exhibited greater transcriptional changes in *P. rapae*-attacked tissue, namely *ORA59, ERF5*, and *AtERF*, all occurring at 15 min after treatment (*p*-value < 0.08). Every gene measured except *MYC2*, which is a JA-responsive gene, responded more strongly to *S. exigua* than to *P. rapae* at a given time point. This was especially true for *ERF104, ERF8, PR4, PR3*, and *ERF11*, whose expression increased in response to *S. exigua*, but declined in response to *P. rapae*. This suggests that these genes are important for the perception of, and response to, *S. exigua*, but not *P. rapae*. When gene expression levels were clustered using Cluster 3.0 (Eisen et al., 1998) by time point and treatment, most *S. exigua* (“Spod”) treatments clustered separately from *P. rapae* (“Pieris”) treatment, except in the case of *MYC2* and *PR4*, indicating that defense gene responses to the two chewing insects are markedly different (Figure 5).

In our experiment with *S. exigua*, we observed several instances where gene expression was increased in control plants, especially during the initial time points, suggesting that thigmotropic stimuli while placing cages (without insects) on the control plant may have contributed to elevated gene expression. We also found that the starting control levels for JA in the *S. exigua* bioassay were higher than in the *P. rapae* assay (Figure 2). We conducted an experiment to determine whether cages put on plants elicited similar patterns on the expression of *ERF8, ERF11, PDF1.2*, and *Thi2.1* as those found in our insect experiments. Our results suggest that touch may be a small contributing factor as gene expression in untouched plants was less than in touched plants, but the transcriptional response elicited by thigmotropic stimulation was not enough to explain the large expression changes in the insect experiments (Supplementary Material, Figure 2). It remains possible that the frequent movement typical of *S. exigua* larvae, which is not shown by *P. rapae* larvae (McCartney, 2007) produced the greater background.

### Insect feeding and glucosinolate assays

**Figure 6:**
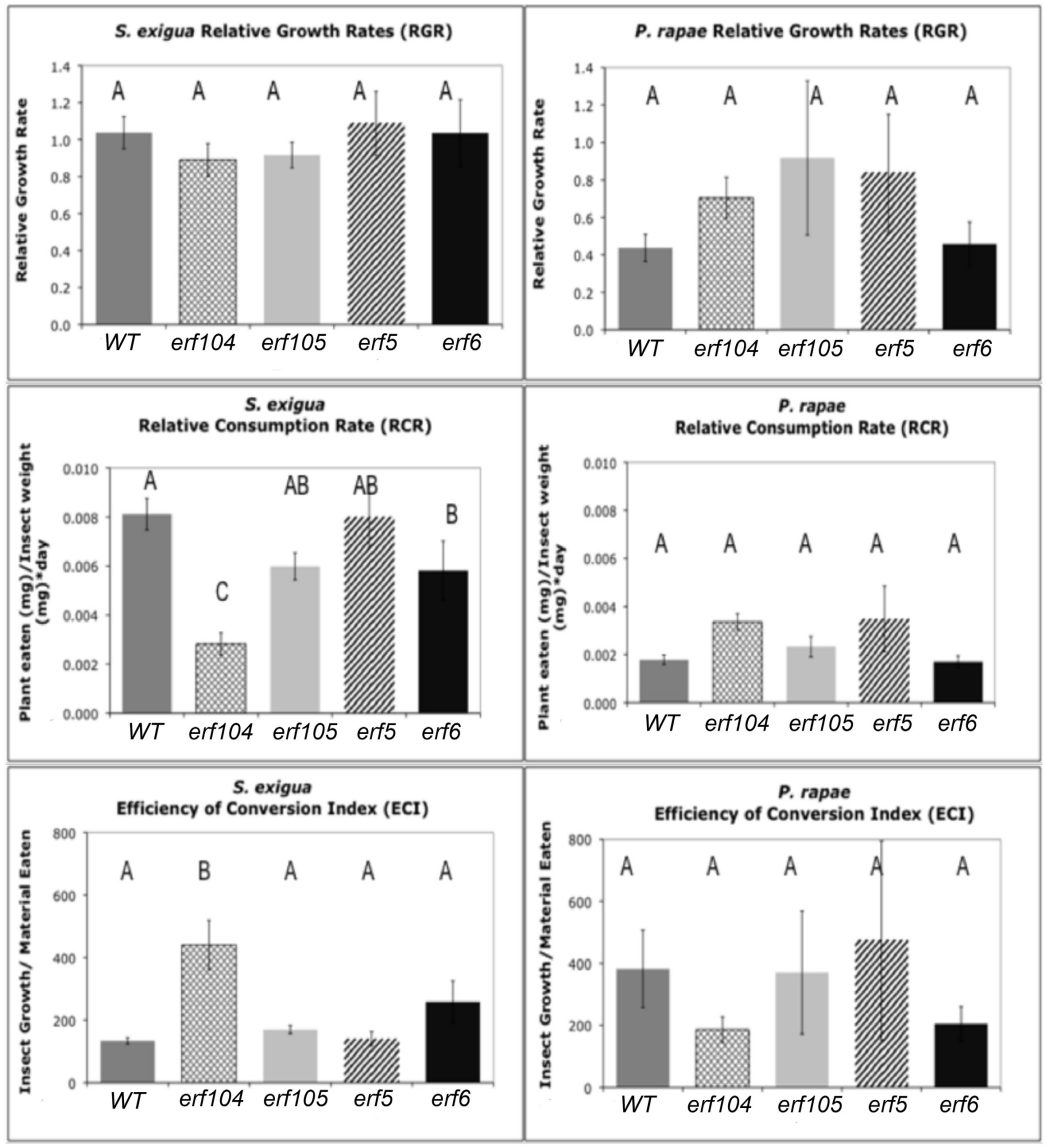
***S. exigua and P. rapae* relative growth rates (RGR), relative consumption rates (RCR), and efficiency of conversion of ingested food index (ECI) on WT and *erf* mutant plants**. RGR represents the total weight gained by an insect relative to its initial weight and total feeding time. RCR is calculated as the total material (mg) eaten divided by initial insect weight (mg) multiplied by the feeding period (days). ECI is calculated as the difference in insect mass before and after the feeding assay divided by the total material (g) eaten. Sample sizes differed between treatments and ranged between 11 and 31 insects. Error bars represent standard errors of the mean. Letters above columns represent *post-hoc* Tukey values after using the GLM model in SAS. Different letters indicate significant differences between genotypes *p* < 0.05.

To assess the role of ERFs in resistance to insects, we conducted no-choice feeding assays with both *P. rapae* and *S. exigua* on WT, *erf104, erf105, erf5*, and *erf5 Arabidopsis* genotypes. As expected, *P. rapae*, which is adapted to feeding on glucosinolate-containing plants, maintained similar RCR, RGR, and efficiencies of conversion of ingested food on all the genotypes (Figure 6). In contrast, *S. exigua* had significantly lower RCR on *erf104* and *erf6*. Although higher specific leaf masses can cause lower RCR because there is more nutrition per unit volume of leaf consumed, this was not the case here because the specific leaf masses did not differ statistically among the genotypes (data not shown). Despite eating significantly less of *erf104* and *erf6, S. exigua* growth rates did not differ among genotypes. This compensatory feeding to maintain a constant growth rate is a common behavior of *S. exigua*. The quality of the *erf* mutants as food for growth also differed for *S. exigua*: ECI was significantly higher on *erf104* than on the other mutants and WT.

The ability of plants to respond to insect feeding by increasing indolyl glucosinolates did not depend on functional ERFs; both *S. exigua* and *P. rapae* feeding increased concentrations of indolyl glucosinolates in the youngest leaves in all genotypes (Supplementary Material, Table S5). In contrast, changes in aliphatic glucosinolates were heterogeneous. When data from all genotypes were combined, there was a significant correlative relationship between total indolyl GS levels and RCR that was negative for *S. exigua* and positive for *P. rapae*, consistent with their tolerance for GS; however in neither case was the relationship strong. Within genotypes, there were only 5 significant relationships between GS levels and RCR and all but one were all positive correlations.

## Discussion

We examined the transcriptional reprogramming of *Arabidopsis* genes encoding TFs elicited by 2 different chewing herbivores or wounding. We identified 141 TF genes that were differentially expressed in at least one treatment. TFs are often points of cross-talk between signaling pathways and have been shown to be important for responses to biotic stresses, including herbivory (Li et al., 1999; Abe et al., 2003; Lorenzo et al., 2003; Fernandez-Calvo et al., 2011; Schweizer et al., 2013). The TF gene expression profiles differed considerably between responses to the two caterpillars, suggesting that responses to different stimuli are shaped by transcriptional activation of stimulus-specific TFs, as are responses to wounding.

### Similarities in arabidopsis responses to chewing caterpillars and wounding

Using a microarray analysis, we found that expression of JAZ, WRKY, and NAC TFs was elevated by both unrelated insects and wounding. This suggests a significant transcriptional role for them after diverse biotic stresses. Many genes in these families were up- or down-regulated across most caterpillar and many wounding treatments. WRKY, NAC, and Zinc-Finger TF induction after *Spodoptera littoralis* feeding was also observed by Schweizer et al. (2013), who suggested a JA-independent role of these TFs in plant defense. Both *S. exigua* and wounding, but not *P. rapae* elicited genes in the C2H2 gene family, including *ZAT10* and *ZAT5*. *ZAT10* modulates a plant's ability to adapt to heat, salinity, and osmotic stress (Mittler et al., 2006) indicating both treatments may be triggering similar abiotic stress-related signaling. However, wounding and insect-induced genes with the most consistent and widespread transcriptional response in our study belonged to the JAZ (ZIM) family, which are important transcriptional regulators in the JA-response. Other studies have reported significant JAZ transcription after wounding, *P. rapae, S. exigua*, or JA treatments (Reymond et al., 2004; DeVos et al., 2005; Chung et al., 2008). JAZ proteins are key regulators of the JA-signaling pathway (Chini et al., 2007; Thines et al., 2007; Fernandez-Calvo et al., 2011) and have been found to be activated in response to *Malacosoma disstria* feeding on poplar (Major and Constabel, 2006), and *S. exigua* feeding on *Arabidopsis* (Chung et al., 2008). These genes may not be involved in differential responses to attack by specific insects, but appear to play a critical role in generalized biotic stress or wound signaling.

Transcriptional factor gene expression was attenuated after wounding in comparison to insect feeding. However, we observed TFs known to play a role as DAMPs including WIPK (Reviewed by Heil and Land, 2014, this edition) and several ZATs (Mittler et al., 2006). The up-regulation of JA-regulated genes, such as JAZ (Chung et al., 2008) and NAC TFs (reviewed by Bu et al., 2008; reviewed by Wasternack and Hause, 2013) suggest a mechanism for the plants' ability to perceive wound damage and trigger responses similar to herbivory. Interestingly, only wounding induced the expression of LOB TFs. LOBs encode a diverse, plant-specific class of proteins that control new growth during root, leaf, shoot, and xylem development (Shuai et al., 2002; Soyano et al., 2008). Fan et al. (2012) showed LOBS to critical players downstream of auxin signaling during callus formation. Taken together, this suggests that LOBs could play a major role in plant recovery or organ regeneration after wounding, but not after herbivore-related damage.

### The major differences in plant responses to the two caterpillars involved the regulation of ERF transcription factors

We observed dramatic differences between the expression profiles of AP2/ERF TFs that were either elicited by either *P. rapae* or *S. exigua* when we conducted both microarray analysis and qRT-PCR. AP2/ERF TFs comprise about 120 members (Nakano et al., 2006), are exclusive to plants, and consist of ERF or B3 DNA binding domains and several sub-families including AP2 and RAV (McGrath et al., 2005). Many ERFs are responsive to the hormones ET and JA (Lorenzo and Solano, 2005; Pre et al., 2008), although individual gene responses to either ET or JA can differ. For example, expression of *ERF1* is compromised in both *coi1* and *ein1* plants (Berrocal-Lobo et al., 2004), which are deficient in JA- and ET-signaling respectively, and the expression of *ERFs* is rapidly induced by exogenous application of both hormones in WT plants (Brown et al., 2003). Fujimoto et al. (2000) showed that ERFs directly activate the transcription of defense-related genes such as *PDF1.2*, B-chitinase (*PR3*), and Hevein-like protein (*PR4*) by binding to GCC-boxes in their promoters. Alternatively, Lorenzo et al. (2004) found the JA-inducible gene *AtMYC* acts to repress the expression of ERFs, while activating other wound-responsive genes such as *VSP2, Thi2.1*, and *LOX3*. Although our subsequent qPCR experiments found some of these ERF TFs to be affected by both caterpillars, we found *TINY2*, to be specifically up-regulated by *P. rapae* whereas *ORA47, SIMRAP2.4, ERF11*, and *ERF104* were only up-regulated by *S. exigua* (Table 1). Each of these genes appears to be important in plant stress responses. For example, *TINY2* transcription increases in response to ABA, drought, salt, cold, wounding, and SA treatment (slightly), but not ethylene (Wei et al., 2005). *ORA47* expression was increased by insect regurgitant from *T. ni* larvae (Walley et al., 2007) and its over-expression increased the expression of the wound-responsive *VSP2* but not *LOX3* (Wang et al., 2008). The RAP2.4 homolog SIMRAP2.4 is responsive to JA, wounding, heat, and water stress and functions in osmoregulation (Walley et al., 2007; Wang et al., 2008; Rae et al., 2011). *ERF11* is an ethylene-inducible transcriptional repressor with an *EAR* motif (Yang et al., 2005) and is highly induced by chitin treatment (Libault et al., 2007), MeJA application and *Alternaria brassicicola* infection (McGrath et al., 2005). *S. exigua* also elicited the expression of *ERF104*, which is a MAPK6 target required for FLG22-induced ET signaling (Bethke et al., 2009). Plants over-expressing *ERF104* had increased transcripts of pathogenesis-related genes that are not induced by *ERF1* activation or JA and ET treatment, so *ERF104* signaling may represent a novel TF response specific to insect attack. We found that *S. exigua* larva that fed *erf104* knock-out mutant *Arabidopsis* plants in no-choice assays did not have reduced growth rates, but did consume less plant mass than other genotypes, including Col-0 WT, *erf5, erf6*, and *erf105* (Figure 6). However, mutant *erf104* plants did not have reduced glucosinolate production (Table S5). This suggests that ERF104 may be a negative regulator of plant defenses and knocking out the gene confers a resistance in *Arabidopsis* plants in a GS-independent manner. Several ERFs (*ERF11, ERF3*, and *ERF4)* contain an *EAR* motif that functions in negative regulation of ethylene-responsive genes via the GCC box (Fujimoto et al., 2000; Brown et al., 2003; McGrath et al., 2005). However, ERF104 does not appear to contain this domain. Interestingly, all four of the ERF TFs uniquely up-regulated by *S. exigua* were found to be highly chitin-responsive by Libault et al. (2007) and may suggest a role in *Arabidopsis*'s differential hormonal and transcriptional response to *S. exigua* vs. *P. rapae* feeding.

### Differences in hormone signaling after caterpillar feeding

Signaling after herbivory involves the crosstalk among JA, ET, SA and other hormones as well as the regulation of TFs and defense related genes (Reviewed by Reymond et al., 2004; Zhu-Salzman et al., 2004; Mewis et al., 2006; Vogel et al., 2007; Wasternack and Hause, 2013). In this study we show that herbivory by two different insects elicits increases in both ET and JA, but not SA. However, the timing of ET and JA responses and the total concentrations induced by the insects were different. In most cases, *S. exigua* elicited stronger, and often earlier, responses, which may shape downstream responses. This is highlighted by the differential expression of ERF TFs and PR genes in response to insect feeding.

Increased ethylene emissions after insect herbivory are well-documented (for review see Von Dahl and Baldwin, 2007). In our study, ethylene production in *Arabidopsis* plants after *S. exigua* attack occurred as a rapid burst and peaked after 1 h (Figure 1). ET levels continued to remain above control levels until 6 h, after which they attenuated. Conversely, *P. rapae* feeding did not induce ET levels that were significantly different from controls until after 2 h, and they remained elevated throughout most of the time course. Our results suggest that ET could serve as an important signal in defense responses to *S. exigua* as well as *P. rapae*, and that the timing of peak ethylene production may be crucial to organizing different down-stream responses to each attacker. These results are consistent with these species' feeding behaviors. *S. exigua* changes feeding sites and/or plants at least every hour, while *P. rapae* larvae may feed on the same leaf for up to a day (McCartney, 2007).

It is well-known that JA production is an important component in plant defense responses, especially after wounding or herbivory. Levels of JA as well as the pre-cursor oxylipins OPDA and dnOPDA gradually increased over a 24-h time course after *P. rapae* feeding (Reymond et al., 2004). DeVos et al. (2006) reported an increase in JA production after *P. rapae* feeding that peaked at 48 h after feeding. Herbivory by *S. exigua* also increased JA levels in *Zea mays* (Schmelz et al., 2003). In this study we found that both insect species increased the production of JA at levels that were significantly different from controls at early time points (*S. exigua*, 30 min; *P. rapae*, 15 min) and which then tapered off after 24 h. Our results suggest that in response to *S. exigua* and *P. rapae*, the timing and ratios of ET and JA may comprise a regulatory mechanism for differential response.

### Defense gene expression after insect feeding

Gene expression of *ERF* TFs and defense-related genes was very different in response to the two insect treatments most likely as a result of varied hormone signaling. *S. exigua* feeding activated the transcription of ERF TFs. Fold change increases in *ERF4, ERF104, ERF8, SIMRAP2.4, ORA59, ERF5, ERF105, ORA47, ERF1, and ERF11* were significantly higher in *S. exigua* treatments. In fact, in only 2 cases, ORA59, and *ERF1* at 15 min, is gene expression significantly higher in *P. rapae*-treated plants. This difference is particularly notable in the expression of *ERF104* and *ERF11*, which are significantly increased by *S. exigua*, and often repressed by *P. rapae* at these time points. Furthermore, *ERF1*, which was also chitin-responsive, is increased by *S. exigua*, but not by *P. rapae* after 6 h.

We observed a similar pattern with the defense-related genes we analyzed. *S. exigua*, but not *P. rapae* elicited the increased expression of *PR3, PR4 (HEL), PDF1.2, LOX3*, and *Thi2.1*, which are JA-responsive genes (Lorenzo et al., 2003, 2004; Koornneef and Pieterse, 2008). In fact, except for LOX3, during the various time points, *P. rapae* down-regulated the expression of these genes compared to *S. exigua*. DeVos et al. (2005) also found that *P. rapae* did not significantly increase *PDF1.2 or PR4 (HEL)* transcription, although an increase in PDF1.2::GUS activity at the periphery of *P*.-damaged tissue was seen. Although both insects elicited the production of JA and ET, *P. rapae* did not increase the transcription of these JA-inducible genes, suggesting that *P. rapae* may be suppressing defense-related signaling. In our study, hormonal and transcriptional responses to *S. exigua* were consistently increased while responses to *P. rapae* were attenuated or in some cases, absent. This, in conjunction with significant JA induction and rapid ET elicitation induced by *S. exigua* vs. *P. rapae* suggests that *P. rapae* may be suppressing host responses or evading detection compared with *S. exigua*. When compared to other biotic stresses, an attenuated transcriptional response in *Arabidopsis* after *P. rapae* attack has previously been reported. For example, Reymond et al. (2000) found that wounding induced far more genes than *P. rapae*, including water-stress related genes and suggested that this may be due to a feeding strategy that reduces overall leaf damage. In a similar study, Bodenhausen and Reymond (2007) found comparable trends with *P. rapae* feeding on Arabidopsis *coi1-1* plants. By using a stress gene-specific microarray, the authors observed more SA-related and disease resistance genes transcribed in the *coi1-1* mutants than in WT plants after *P. rapae* feeding, suggesting a putative mechanism for JA-dependent gene suppression. Interestingly, the same response was not seen after *Spodoptera littoralis* feeding. In our study, we found that this potential suppression of responses is not likely due to an accumulation of SA, but could be a by-product of weakened ET and JA elicitation compared to *S. exigua*.

The cross-talk among the ET, JA, ABA, and SA signaling-pathways in response to pathogens and insects is complex and involves antagonisms between the ABA/JA branch via MYC2 and the ET/JA branch via ERFs (reviewed by Wasternack and Hause, 2013). Both of these branches require activation of JAZ proteins, which were up-regulated in both insect treatments. One hypothesis posed by Verhage et al. (2011) states that *Arabidopsis* plants quickly perceive *P. rapae* feeding and suppress responses by activating a JA-dependent MYC2 pathway vs. a JA/ET-dependent ERF pathway. A previous study by DeVos et al. (2006) found that wounding + *Pieris* OS suppressed *PDF1.2* likely through the ABA-activation of *AtMYC2*. Recently, Vos et al. (2013) found that induction of AtMYC2 by *P. rapae* is largely controlled by ABA and JA, which act together to prime the plant's defenses against future herbivores. Our results further support the hypothesis that *P. rapae* elicits the ABA/JA-MYC2 pathway, thus by-passing ET/JA-ERF signaling. Conversely, *S. exigua* clearly elicits a rapid JA and ET “burst” (Von Dahl et al., 2007), which most likely has downstream effects on ERF transcription and JA-mediated responses. In many ways, we observed that responses to *S. exigua* were more aligned with pathways activated by necrotrophic pathogens because of its activation of the ERF-branch of the biotic stress response (reviewed by Pieterse et al., 2012).

Interestingly, when Verhage et al. (2011) used *P. rapae* wounding + OS, the ERF pathway was activated. Yet, the application of *S. littoralis* and *Pieris brassicae* OS suppressed several wound-responsive genes in Arabidopsis (Consales et al., 2012), including an ERF-family TF. It is tempting to speculate that something about *Pieris* feeding behavior attenuates wound signaling and that the differences in defense responses, including hormone release and TF transcription, originate at the feeding site. This suggests that an insect's ability to elicit responses divergent from wound responses may be a key element of host plant specialization. In our comparison and results of others, responses to *S. exigua*, an herbivore with a very broad diet, more closely resemble responses to necrotophic pathogens than do responses to the more specialized *P. rapae*. We observed that at the whole plant level, *P. rapae* feeds in one location on the edge of a leaf and continues to remove tissue from that site. Conversely, *S. exigua* eats small amounts throughout the plant, creating several small holes throughout the leaves and distributing their damage across the leaf and/or plant (McCartney, 2007). In a previous study (Rehrig et al., 2011), we found that herbivory by *S. exigua* was particularly distressing to *Arabidopsis* plants and was most likely affecting both primary and secondary metabolism. The similar results reported here suggest that the trauma inflicted by *S. exigua* may be a due to a combination of HAMPs and DAMPs elicited after feeding. Furthermore, due to the elicitation of ET, JA, and chitin-responsive genes, *S. exigua* feeding activates responses that are more similar to microbe-associated molecular responses (MAMPs) than herbiviory.

## Author contributions

Erin M. Rehrig—Prepared manuscript, including tables and figures, analyzed and interpreted data, conducted qRT-PCR and all insect rearing and bioassays. Heidi M. Appel—Edited manuscript, interpreted data, managed microarray project, oversaw insect and plant growth, co-authored funding for project. A. Daniel Jones—Conducted UPLC-MS/MS analysis on JA, SA, and JA-IL on plant samples, edited manuscript and provided critical feedback. Jack C. Schultz—Edited manuscript, assisted with the analysis and interpretation of data, conducted statistics, procured funding for project

### Conflict of interest statement

The authors declare that the research was conducted in the absence of any commercial or financial relationships that could be construed as a potential conflict of interest.
