# Supplementary material for: Roles for jasmonate- and ethylene-induced transcription factors in the ability of Arabidopsis to respond differentially to damage caused by two insect herbivores
Source: Front Plant Sci. 2014 Aug 19;5:407. doi: 10.3389/fpls.2014.00407 (PMC4137388; doi:10.3389/fpls.2014.00407)
Supplement: Supplementary file 1 [file DataSheet1.DOCX]

**Supplemental Figures and Experiments:**


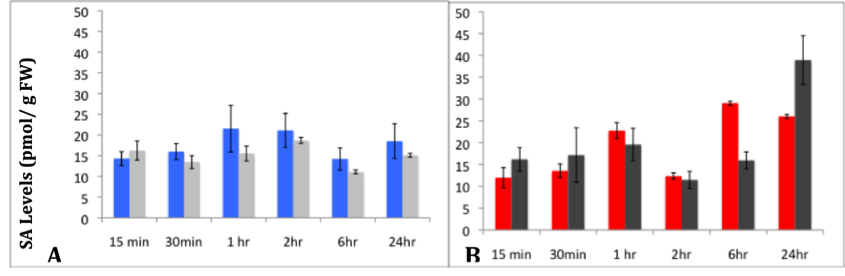


**Figure S1:** Salicylic Acid (SA) production in WT Arabidopsis plants after S. exigua (A) or P. rapae (B) feeding over a 24hr time course- SA was measured as pmol/g fresh weight. Blue bars represent S. exigua feeding; light gray bars represent controls in the S. exigua experiment. Red bars in (B) represent P. rapae treatment, dark gray bars represent controls in the P. rapae experiment. Error bars are standard errors of the mean


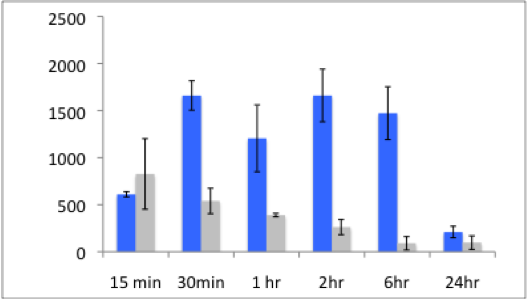


JA (pmol/g FW)

*

*

*


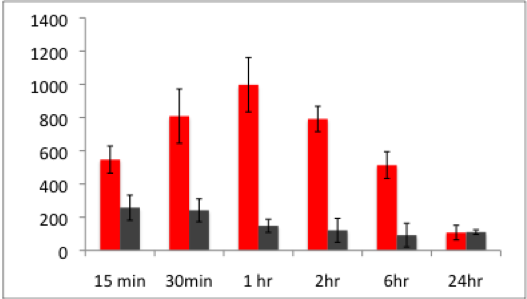


*

*

*

*

*

**A**

**B**

**Figure S2:** **Jasmonic Acid levels in WT *Arabidopsis* plants after *S. exigua* (A) or *P. rapae* (B) feeding over a 24hr time course-** JA was measured by HPLC-MS as pmol/g fresh weight. Blue bars represent *S. exigua* feeding; light gray bars represent controls in the *S. exigua* experiment. Red bars in (B) represent *P. rapae* treatment, dark gray bars represent controls in the *P. rapae* experiment. Asterisks represent data points that are significantly different than controls (p<0.05). Error bars are standard errors of the mean.


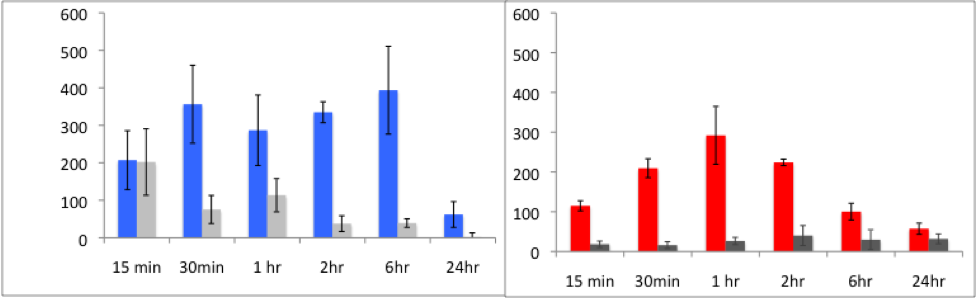


**JA-IL (pmol/ g FW)**

*

*

*

**A**

**B**

*

*

*

*

*

**Figure S3:** **Jasmonic Acid-isoleucine (JA-IL) levels in WT *Arabidopsis* plants after *S. exigua* (A) or *P. rapae* (B) feeding over a 24hr time course-** JA-IL was measured as pmol/g fresh weight. Blue bars represent *S. exigua* feeding; light gray bars represent controls in the *S. exigua* experiment. Red bars in (B) represent *P. rapae* treatment, dark gray bars represent controls in the *P. rapae* experiment. Asterisks represent data points that are significantly different than controls (p<0.05). Error bars are standard errors of the mean.

**Supplementary Experiment:**

*Touch Experiment:* Because we observed some unexpected peaks in gene expression (*ERF4, ERF8, ERF11, PDF1.2*) in control samples after 15 min and 30 minutes, we conducted a simple experiment to measure the expression of these genes in plants given only a cage/touch treatment vs. no contact. Six-week old plants grown in the exact same conditions as described above were used. To mimic the caged controls in the original experiments, we placed cages on 4 middle-rosette leaves, occasionally manually adjusted them for 40 minutes, and removed them in 2, 20-minute increments. Control plants received no cages and were minimally disturbed so not to induce a touch response. Plant tissue was harvested for RNA/gene expression analysis 15 or 30 minutes later as described in the Materials and Methods.

**Figure S4: Gene expression of ERFs and defense genes in response to caging (touch).** Black bars represent control plants (no touch) while gray bars represent samples that were caged and handled to mimic the control plants in the insect experiments. Error bars are +/- standard error of the means (n=3). Astericks represent significant induction of gene expression as determined by ANOVA and *post-hoc* Dunnett’s tests.

*

*

*

*


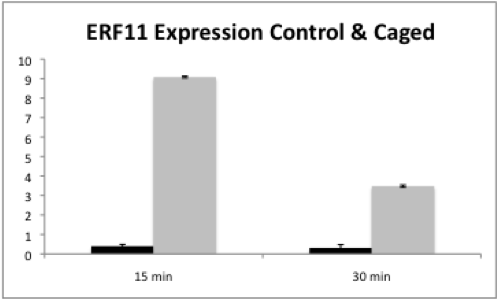

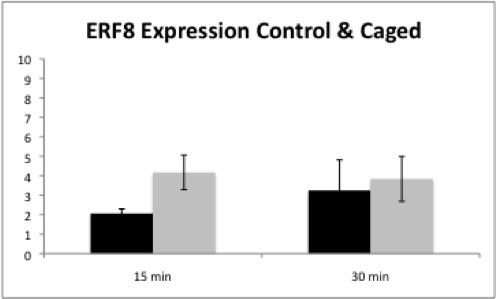

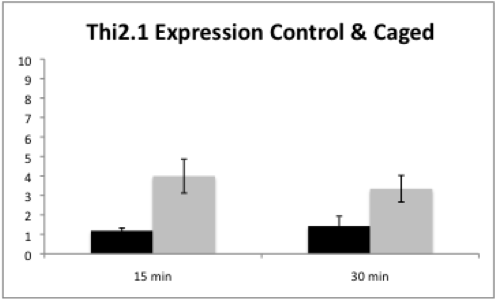

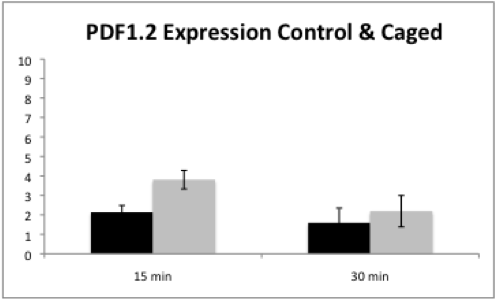


*

*

**Figure S5: Constitutive and induced Indolyl and Aliphatic Glucosinolate levels in WT and *erf* plants in *S. exigua* and P*.rapae* bioassays-** Using HPLC-MS, constitutive levels of total indolyl (A, B) and aliphatic (C, D) GSs were determined in control plants and induced levels were measured after *S. exigua* (A, C) or *P. rapae* (B, D) treatments. Error bars represent standard errors of the mean. Letters above columns represent post-hoc Tukey values. Different letters indicate significant differences between genotypes. Lowercase letters represent Tukey values for constitutive GS levels, while capital letters indicate differences between induced GS levels. Asterisks represent significant differences between constitutive and induced GS levels within each genotype.


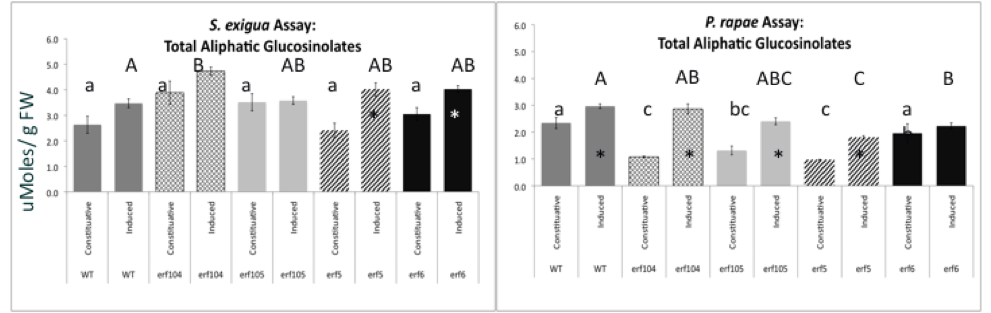

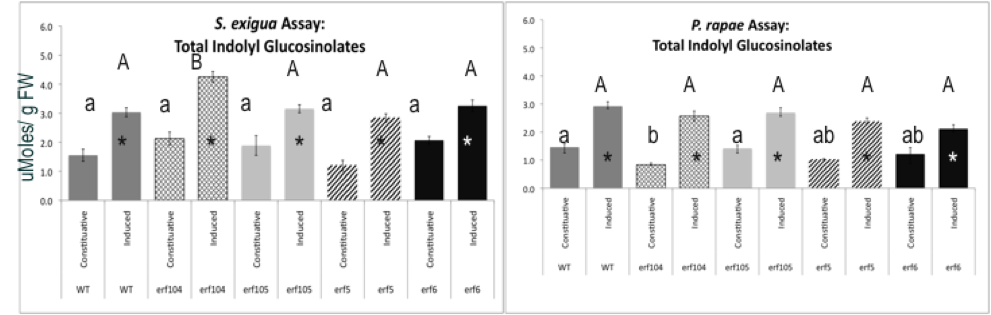


**C**

**D**

**A**

**B**


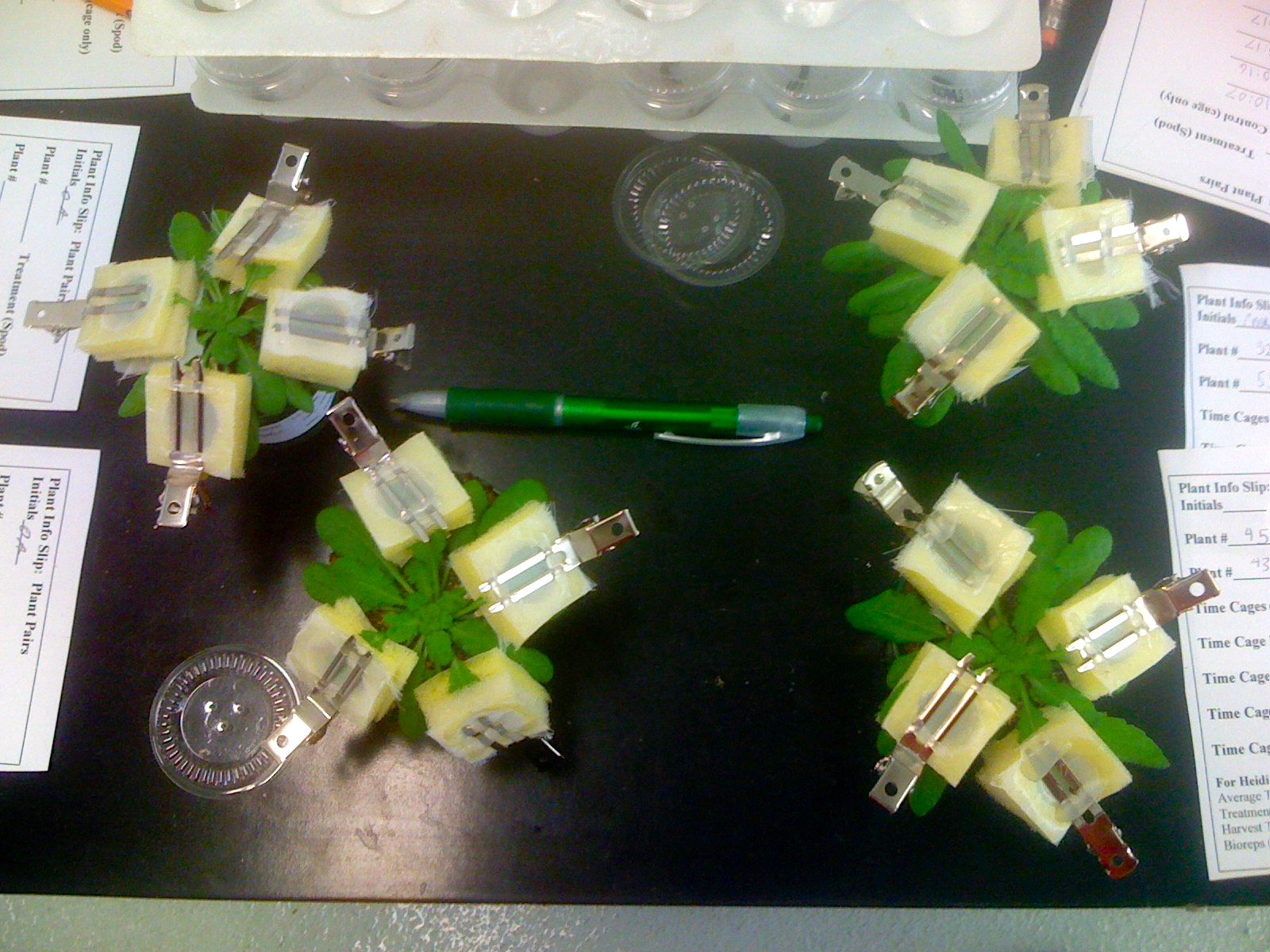

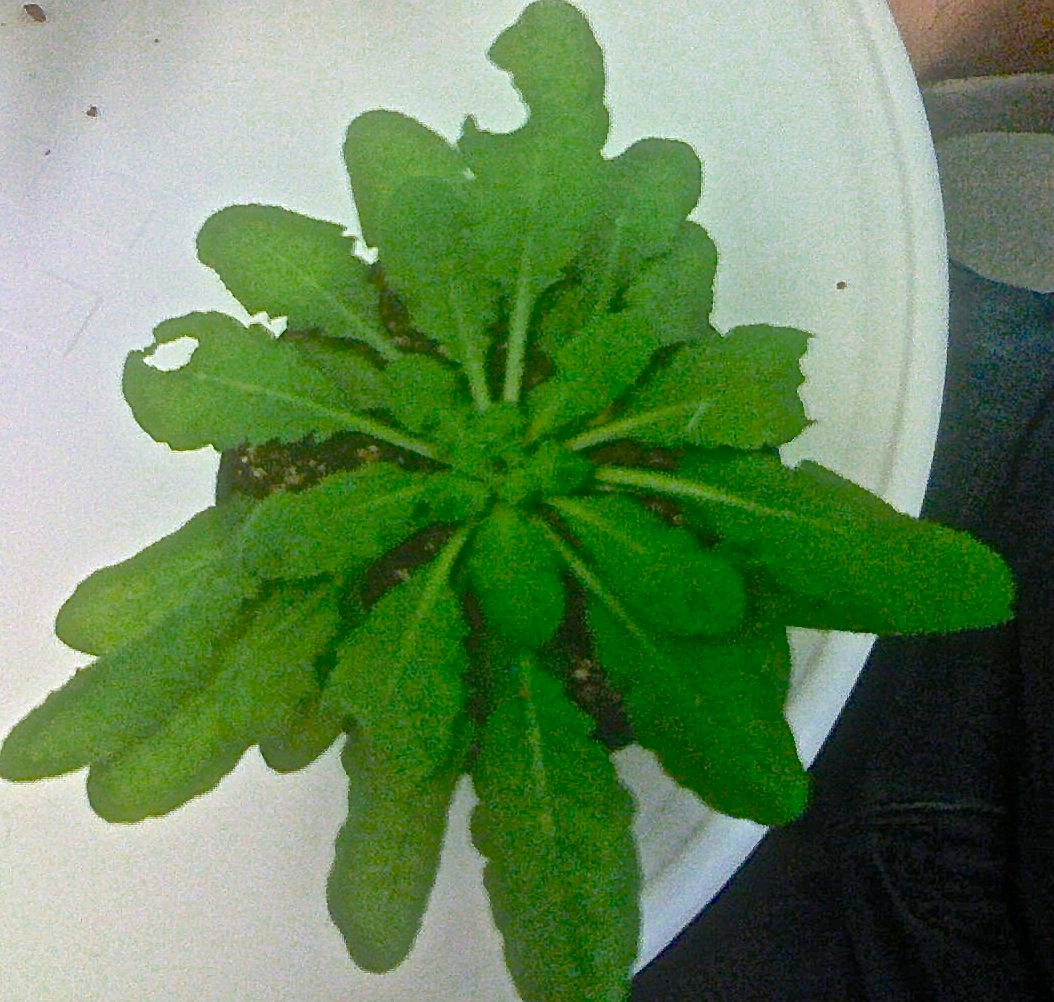


A

B

**Figure S6A & S6B:** Photos of plants with customized cages for insects. A. Control plants received cages without insects. B. Photo showing 20-30% insect damaged leaves (approximately 20 minutes of feeding time).
